# The memory B cell response to influenza vaccination is impaired in older persons

**DOI:** 10.1016/j.celrep.2024.113745

**Published:** 2024-01-28

**Authors:** Alice R. Burton, Stephane M. Guillaume, William S. Foster, Adam K. Wheatley, Danika L. Hill, Edward J. Carr, Michelle A. Linterman

(Cell Reports *41*, 111613; November 8, 2022)

## Main text

In the originally published version of this article, Figure S5 of the online supplementary information did not display correctly. This error was introduced during typesetting and was not identified at the proofing stage. Due to the age of the article, the supplementary information file could not be updated, but the intended Figure S5 can be seen below. This does not change the conclusions of the study.

The authors regret this error.

**Figure S5:**
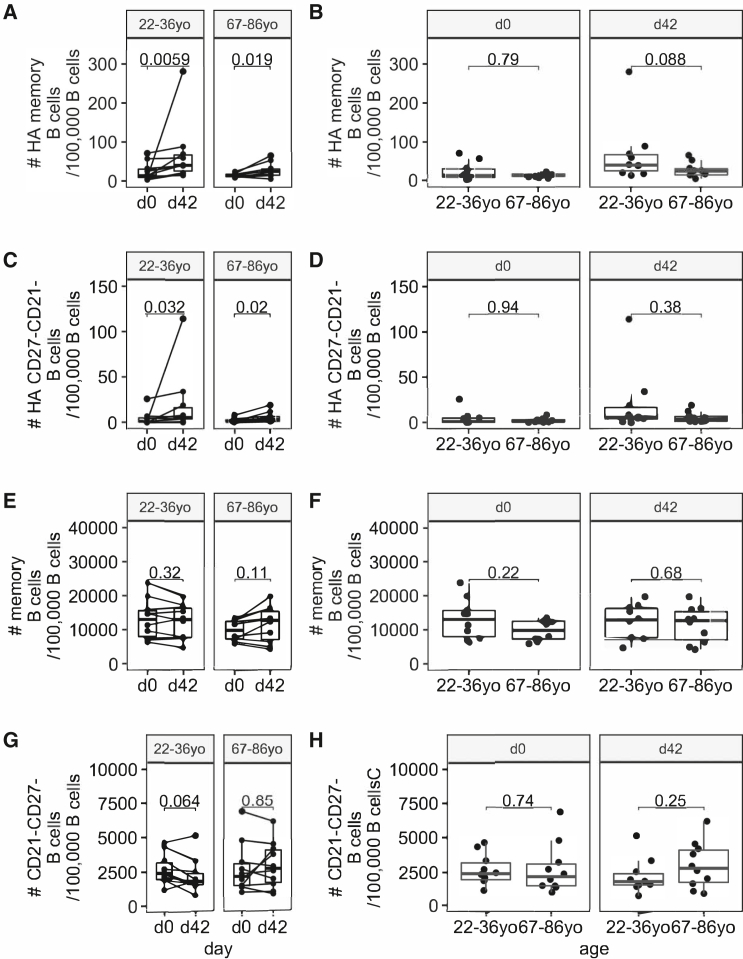
Haemagglutinin specific CD27+ and CD27-CD21- B cell populations, are expanded after vaccination (corrected)

